# Evaluating malformations of the lacrimal drainage system in brachycephalic dog breeds: A comparative computed tomography analysis

**DOI:** 10.1371/journal.pone.0257020

**Published:** 2021-09-07

**Authors:** Sabine Sahr, André Dietrich, Gerhard Oechtering

**Affiliations:** 1 Small Animal Department, Faculty of Veterinary Medicine, University of Leipzig, Leipzig, Germany; 2 Department of Computer Science, Technische Universität Bergakademie Freiberg, Freiberg, Germany; Faculty of Animal Sciences and Food Engineering, University of São Paulo, BRAZIL

## Abstract

**Objectives:**

This study aimed to investigate and compare the anatomical features of the nasolacrimal drainage system (NDS) in three brachycephalic dog breeds with those of normocephalic dogs, taking into account how the NDS was related to the malformed brachycephalic head.

**Animals:**

Fifty-one brachycephalic dogs were examined, comprising 23 Pugs, 18 French Bulldogs, and 10 English Bulldogs. Six normocephalic dogs of different breeds served as a comparison.

**Methods:**

Computed tomographic dacryocystography was performed. Parameters such as length, angulation, and gradient were determined. Crossing of the nasolacrimal duct (NLD) beneath the maxillary canine root, as well as the incidence of an accessory opening, were also analyzed.

**Results and conclusions:**

In all three brachycephalic breeds, the NDS was grossly malformed. We regard this as a further consequence of exaggerated breeding for a short head conformation. While the length of the NLD was substantially reduced by 41 to 57 percent in brachycephalic dogs, their lacrimal canaliculi were two to three times as long as those of normocephalic dogs. Varying parts of the nasolacrimal drainage system followed an inverse direction in short-headed dogs, giving the entire nasolacrimal apparatus an anomalous U- or V-shaped appearance. The NLD exhibited a three to five times steeper alignment in brachycephalic dogs than in normocephalic ones. Obviously, this strong slope did not cause clinical symptoms only because there was an aberrant outflow pathway. The brachycephalic dogs consistently exhibited an accessory opening, through which most of fluid escaped into the posterior nasal cavity instead of through the common route into the nasal vestibule via the nasolacrimal ostia.

## Introduction

Many studies have shown that exaggerated breeding for brachycephaly leads to pronounced malformations in many areas other than the head [1–4], particularly the upper respiratory tract. Furthermore, several structures in the head itself are impaired in both form and function by this pathological head shape. In particular, the abnormalities in the area of the eyes are well documented, and recent studies have shown severe malformations in the middle ear [5–7].

In a previous study [2], a new intranasal surgical procedure for intranasal airway obstruction in animals was introduced. We carried out preliminary investigations using this new approach to determine whether the anatomy of the nasolacrimal drainage system (NDS) would be violated. We found serious deviations from the normal nasolacrimal drainage system anatomy. This prompted us to investigate tear-drainage routes before surgery in a larger group of patients and allowed us to assess surgical resection margins during surgery. The resulting data were used to characterize the NDS in detail. While the literature contains comprehensive information about the anatomical properties of the lacrimal apparatus in normocephalic dogs [8–12], as well as in both normo- and brachycephalic cats [3, 10, 11, 13–15], little is known about the system in brachycephalic dog breeds. Thus, the present study compares the anatomical and functional characteristics of the nasolacrimal apparatus between brachycephalic dog breeds and normocephalic dogs. The study led to several conclusions regarding potentially adverse consequences of brachycephaly on the functionality of the NDS.

## Animals and methods

The study included three brachycephalic dog breeds, Pugs, French and English Bulldogs. They were presented for the diagnosis and treatment of brachycephalic respiratory distress syndrome at the ENT-Unit, Small Animal Department, University of Leipzig, Germany. The owners’ consent was obtained in advance. Dogs with severe conjunctivitis and evidence of dacryocystitis were excluded from the study.

Normocephalic dogs of different breeds served as a comparison. They had been subjected to post mortem computed tomography dacryocystography (CT-DCG) at the University of Leipzig in another study [8]. The images obtained were completely reevaluated for the purposes of the present study.

The CT-DCG complemented the indication-based CT diagnosis of brachycephalic syndrome. This study was carried out in strict accordance with the German Animal Welfare Act. The protocol was approved by the Animal Welfare Committee of the University of Leipzig (Protocol Number: VMF A 19/2012_KTK). All examination was performed under general anaesthesia, and all efforts were made to minimize suffering. Anesthesia was induced intravenously with diazepam (0.5 mg/kg) and levomethadone (0.5 mg/kg). All animals were intubated, and anesthesia was maintained with 2% isoflurane in 50% oxygen [16]. CT-DCG was performed on all dogs using iodinated contrast medium (Iopamidol; Imeron 300M®, Bracco Imaging Deutschland GmbH, 78467, Konstanz). CT studies were carried out using a six-row multislice CT scanner (Philips Brilliance 8000 Mx; Philips Medical Systems, Hamburg, Germany). The dogs were positioned in sternal recumbency using an appropriate positioning aid, with their hard palate parallel to the table [8, 9]. To fill the NDS with the contrast agent, the superior lacrimal canaliculi were cannulated bilaterally using either a 22-gauge or a 24-gauge plastic catheter. To keep the NDS filled with contrast medium until the CT scan was completed, we increased the medium’s viscosity by mixing it with methylcellulose (Methocel® 2%; OmniVision GmbH, 82178 Puchheim, Germany; [13]). Depending on animal size, between one and two milliliters of contrast agent was administered. The CT scans were then performed in a transverse plane with constant parameters (150–200 mAs, 120–140 kV, pitch 0.6, slice thickness: 0.6–1.0 mm). The CT scans of the normocephalic group were carried out in the same manner as part of a previous study in our hospital [8] (CT parameters: 50 or 300 mAs; 120 kV, pitch 0.6–0.9, slice thickness: 0.8 or 2.0 mm). Unlike the brachycephalic group, which were alive at the time of imaging, the normocephalic dogs were examined shortly after death. The image viewing program e-film workstation 2.1 (Merge Healthcare) enabled the CT images to be evaluated in at least three different planes (sagittal, transversal, dorsal) using the bone reconstruction algorithm. Three-dimensional image visualization and various measurements were obtained using an open-source image processing program (ImageJ; National Institutes of Health, USA).

To quantify the course of the entire NDS in the different dog breeds, parameters such as angulation, length and gradient were determined.

**Fig 1:**
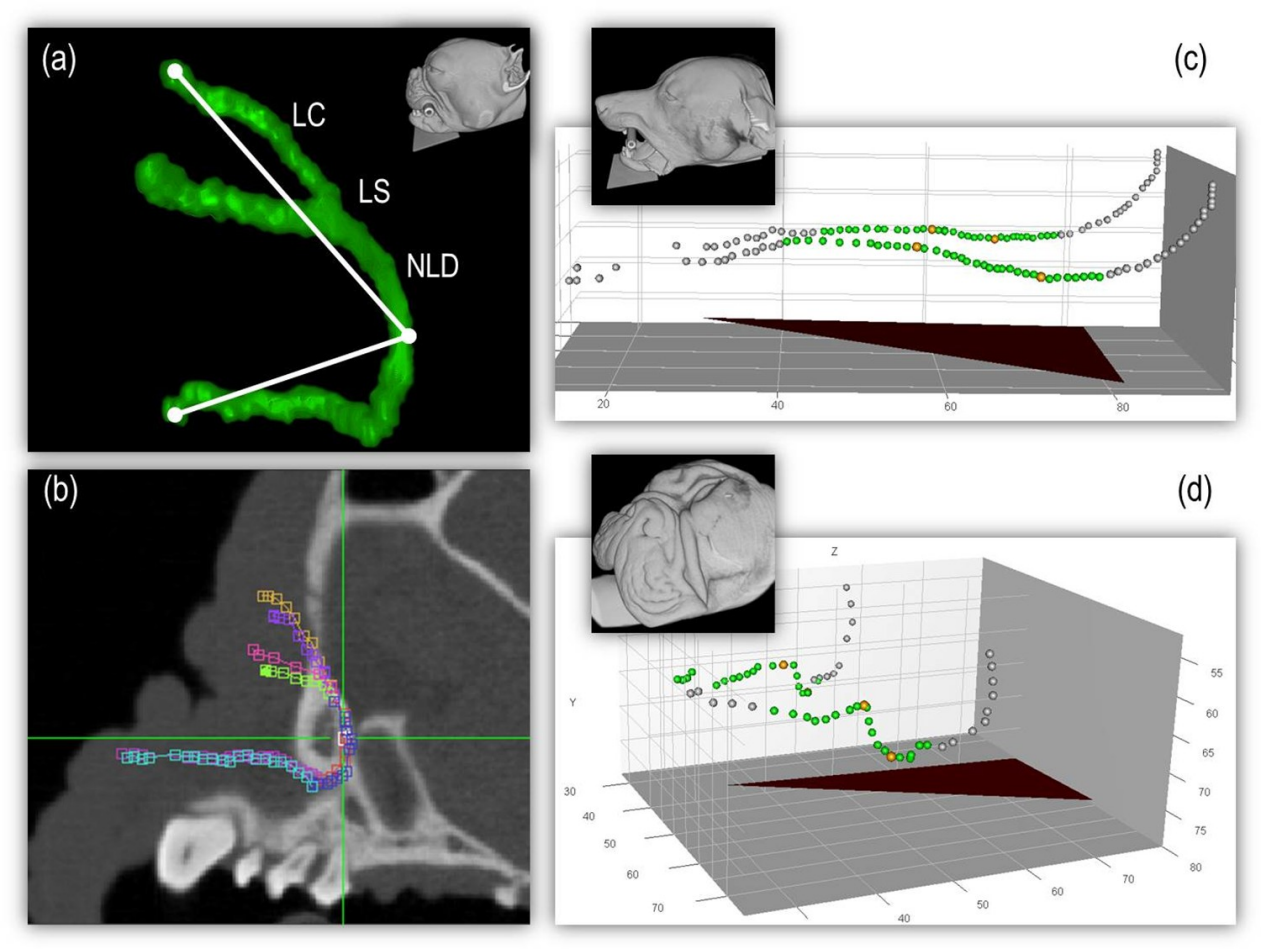
Methods to measure angulation, length, and slope of the nasolacrimal duct (NLD). The images depict the measurement methods used to determine the angulation, length, and slope of the NLD. The viewing perspective is represented by the heads in the picture. (a) Angulation: A 3D illustration of the complete nasolacrimal drainage system (NDS) (unilateral) is shown. Visible are the three points used for angle determination: the upper lacrimal punctum, the caudalmost point of the NLD (vertex), and the nasolacrimal ostium. LC = lacrimal canaliculi; LS = lacrimal sac; NLD = nasolacrimal duct. (b) Length: In a CT sagittal section of a Pug, the different parts of the NDS can be seen (bilateral) marked in different colors. The NDS was marked manually point by point along its entire course using the colored markers shown. The lengths of the individual segments were determined separately. All straight distances between the individual markers were then added to the total length of one segment of the NDS. (c) and (d) Slope: The picture shows the NLD bilaterally (not the entire NDS) in a three-dimensional coordinate system. The coordinates of the NLD are illustrated as dots. The triangle represents the alignment of the hard palate. The green dots show the area included in the measurement. The orange dots represent the lowest and highest points. The grayish dots are outside the area of interest. The gradient was obtained starting from the lowest point of the NLD shortly before leaving the osseous lacrimal canal. The second measuring point was the peak of its middle portion. The lowest and highest points of the NLD were determined in relation to the alignment of the hard palate (triangle), so the results were unaffected by head positioning.

To measure the angulation of the NDS, we used the 3D-Angle Tool in ImageJ (Fig 1A). We determined the angle between the upper lacrimal punctum, the most caudal point of the nasolacrimal duct (NLD; vertex), and the nasolacrimal ostium. Furthermore, we measured the lengths of the distinct parts of the NDS to compare them with the rare and sometimes old data [10, 12]. in the literature (Fig 1B). This particular procedure required a good contrast across the entire NDS, which was achieved using either contrast agent or air. Using the 5D-Viewer in ImageJ, the different parts of the lacrimal apparatus were marked manually along their entire course. Afterwards, all straight distances between the single markers were added up to obtain the total lengths of each NDS segment (e.g. upper canaliculi, NLD). Because the borders of the lacrimal sac are not sufficiently definable, its length was included within the NLD. To quantify the course of the NLD, we determined its gradient in percentage terms. A program was written in *Gnu R 2*.*14*.*1* to calculate the gradient, starting from the lowest point of the NLD, which is usually located shortly before the end point of the bony lacrimal canal (Fig 1C and 1D). The second measuring point is the highest peak of the middle portion of the NLD. The lowest and highest points were determined in relation to the alignment of the hard palate, so the results were unaffected by the head positioning in the CT scanner.

Beyond this, additional characteristics of the NDS were analyzed, 1. whether the NLD crossed below the root of the maxillary canine to reach the nasal cavity, 2. whether there was any accessory opening, and 3. where this was located.

### Statistical analysis

The data were measured separately in each half of the face. In the descriptive statistical analyses, the values of the left and the right side were summed to improve clarity. The Shapiro-Wilk normality test was used to determine normal distribution. Because the data varied between normal and non-normal distributions, exclusively non-parametric tests were applied to identify significant differences between the sample groups (Mann-Whitney U test for independent samples). The relationship between NLD gradient and drainage was examined in the same way (Group subdivision into: drainage yes / no), as was the relationship between NLD gradient and crossing under the maxillary canine roots (Group subdivision into: crossing yes / no). The chi-square test was used to identify relationships between crossing under the maxillary canine and drainage. Drainage was defined as present when the fluid did not completely escape into the posterior nasal cavity through the accessory opening, but followed the common route into the nasal vestibule. All calculations are based on a 95% level of significance. The statistics program Sigma Plot 11.0 (Systat Software, Inc.) was used.

## Results

The present study included a total of 51 dogs (31 males, 20 females) of three brachycephalic breeds, 23 Pugs, 18 French Bulldogs, and 10 English Bulldogs. They were aged between 10 and 108 months (median age: 31 months). More specifically, out of 23 Pugs 14 were males, 9 females, they were 10–105 months old, median age was 38 months, out of 18 French Bulldogs 13 were males, 5 females, they were 10–108 months old, median age was 26 months, and out of 10 English Bulldogs 4 were males, 6 females, they were 11–58 months old, median age was 30 months. Six normocephalic dogs of different breeds served as a comparison. Among them, there was one Beagle, one Rhodesian Ridgeback, one Bearded Collie, one Cairn Terrier, and two mixed-breed dogs; three were males and three females, and they were aged between 20 and 168 months (median age: 148 months).

### Course of the NDS

**Fig 2:**
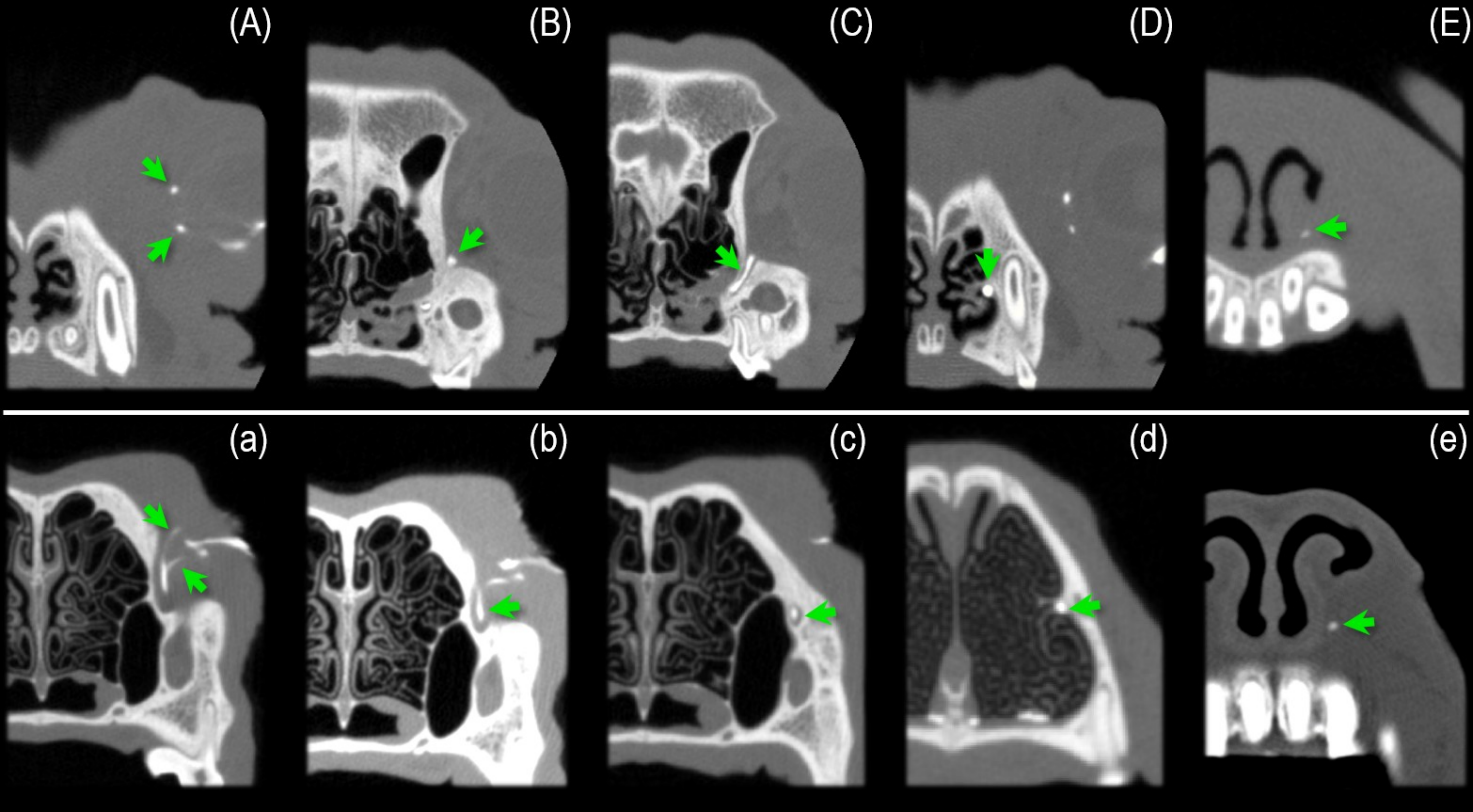
The NDS of a brachy- and normocephalic dog in CT cross-sectional images. The picture illustrates the different, contrast medium-filled parts of the NDS in CT cross-sectional images. Figures (A)—(E) show a French bulldog, (a)—(e) a normocephalic mixed breed dog in comparison. (A) and (a) focus on the lacrimal canaliculi. It is noticeable that the canaliculi in the brachycephalic dog, due to their backward course, are shown in cross-section (punctiform), while in the normocephalic dog they are shown in longitudinal section. (B) and (b) depict the lacrimal sac. While the lacrimal sac in the normocephalic dog appears as a well delineated, drop-shaped structure, in the French bulldog it is clumsy and poorly demarcated. (C) and (c) show the nasolacrimal duct (NLD) within its bony canal. The different course of the NLD is also noticeable. (D) and (d) illustrate the middle portion of the NLD below the basal lamina of the ventral nasal concha. Here the NLD has a larger diameter than the initial part. (E) and (e) show the end portion of the NLD as it runs deep below the mucous membrane just before it emerges from the lacrimal foramen. Please note that in the French Bulldog different parts of the NDS are visible within one CT cross-sectional image. This is never the case with the images of the normocephalic dog. Pictures (A) and (D) show both the canaliculi and the middle portion of the NLD. Image (B) depicts the lacrimal sac and the end of the initial part of the NLD.

**Fig 3:**
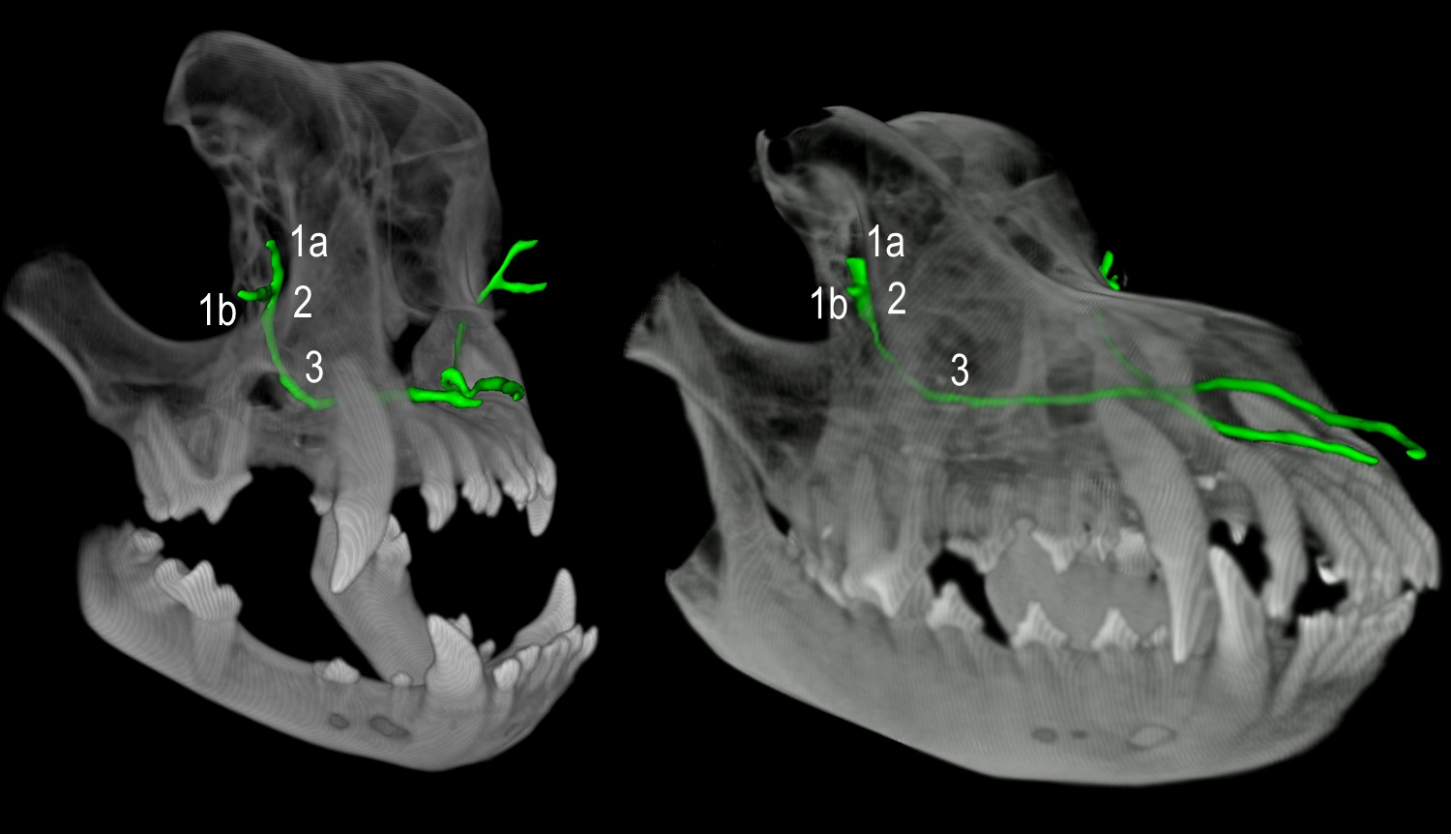
Comparative course of the nasolacrimal drainage system (NDS) in a brachy- and normocephalic dog. The NDS is illustrated three-dimensionally, in green color, inside the skull of an English Bulldog and a Rhodesian Ridgeback. In the English Bulldog, the lacrimal canaliculi and initial part of the nasolacrimal duct (NLD) are directed backwards, the lacrimal canaliculi have much larger dimensions than those in the normocephalic dog, and the NLD has to cross beneath the maxillary canine root to reach the nasal cavity. (1a) upper lacrimal canaliculi, (1b) lower lacrimal canaliculi, (2) lacrimal sac, (3) nasolacrimal duct.

**Fig 4:**
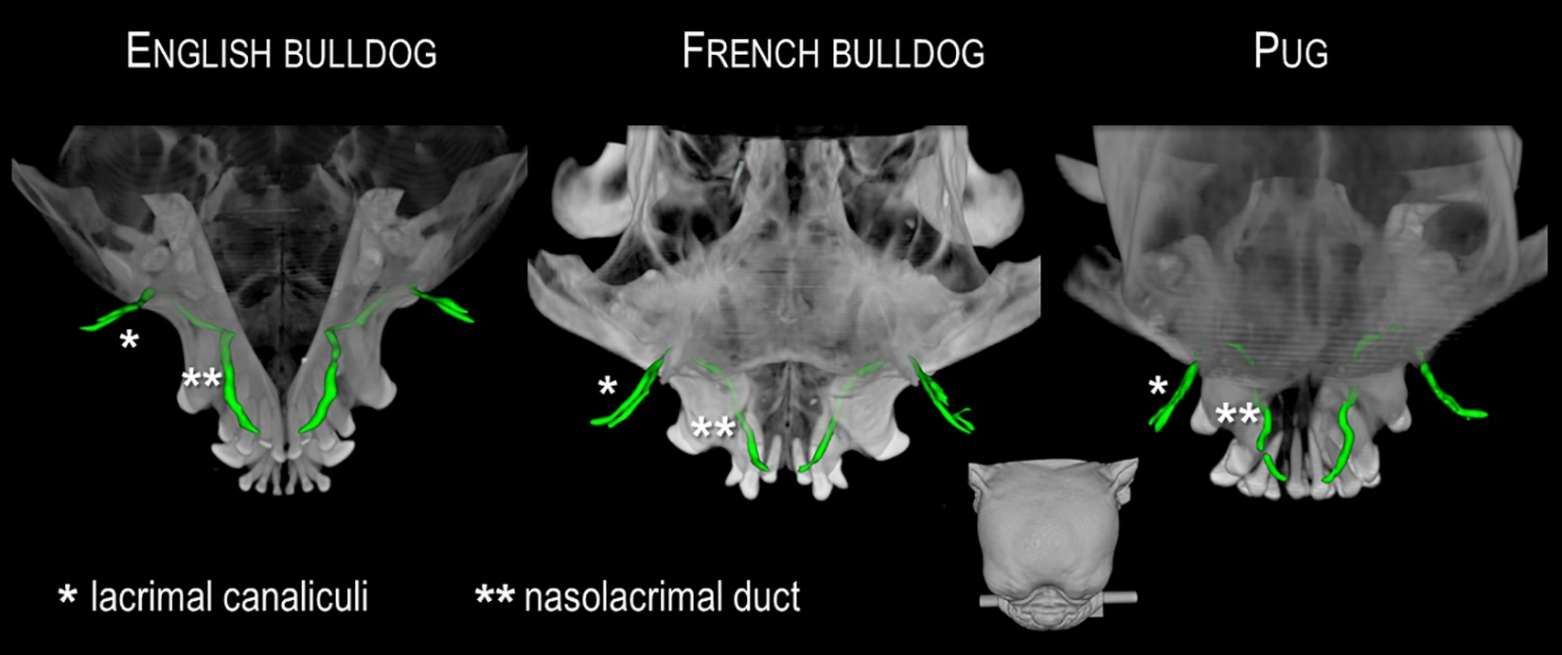
Comparative course of the nasolacrimal drainage system (NDS) in three brachycephalic dog breeds. The NDS is illustrated three-dimensionally, in green color, inside the skull of an English Bulldog, a French Bulldog, and a Pug. The viewing perspective is represented by the head at the bottom of the picture (dorsal view). The backwards orientated parts of the NDS (canaliculi, initial part of the nasolacrimal duct) are smallest in English Bulldogs and largest in Pugs.

The lacrimal canaliculi of normocephalic dogs are rostroventrally orientated, the canaliculi of short-headed ones follow an inverse direction (Figs 2, 3, 4, 7). They are displaced rostrally apparently because the globes have a forward position in these dogs [17]. The adjoining lacrimal sac, which has a characteristic drop-shaped structure in the normocephalic dog, is in all brachycephalic dogs a short, indistinct object that is frequently difficult to identify (Fig 2).

In the brachycephalic dogs, the first segment of the NLD is translocated posteriorly. Varying parts are directed backwards, such as the lacrimal canaliculi. During its way through the bony lacrimal canal, the NLD changes direction and turns rostrally. Immediately after emerging from the bony lacrimal canal, the middle portion of the NLD ascends until it reaches the basal lamina of the ventral nasal concha. This ascent is measurably steeper in short-headed breeds than in normocephalic ones. The further course of the NLD is continuously descending in both normo- and brachycephalic dogs until it ends at the nasolacrimal foramen (Figs 2, 3, 7).

### Ascent of the NLD

**Fig 5:**
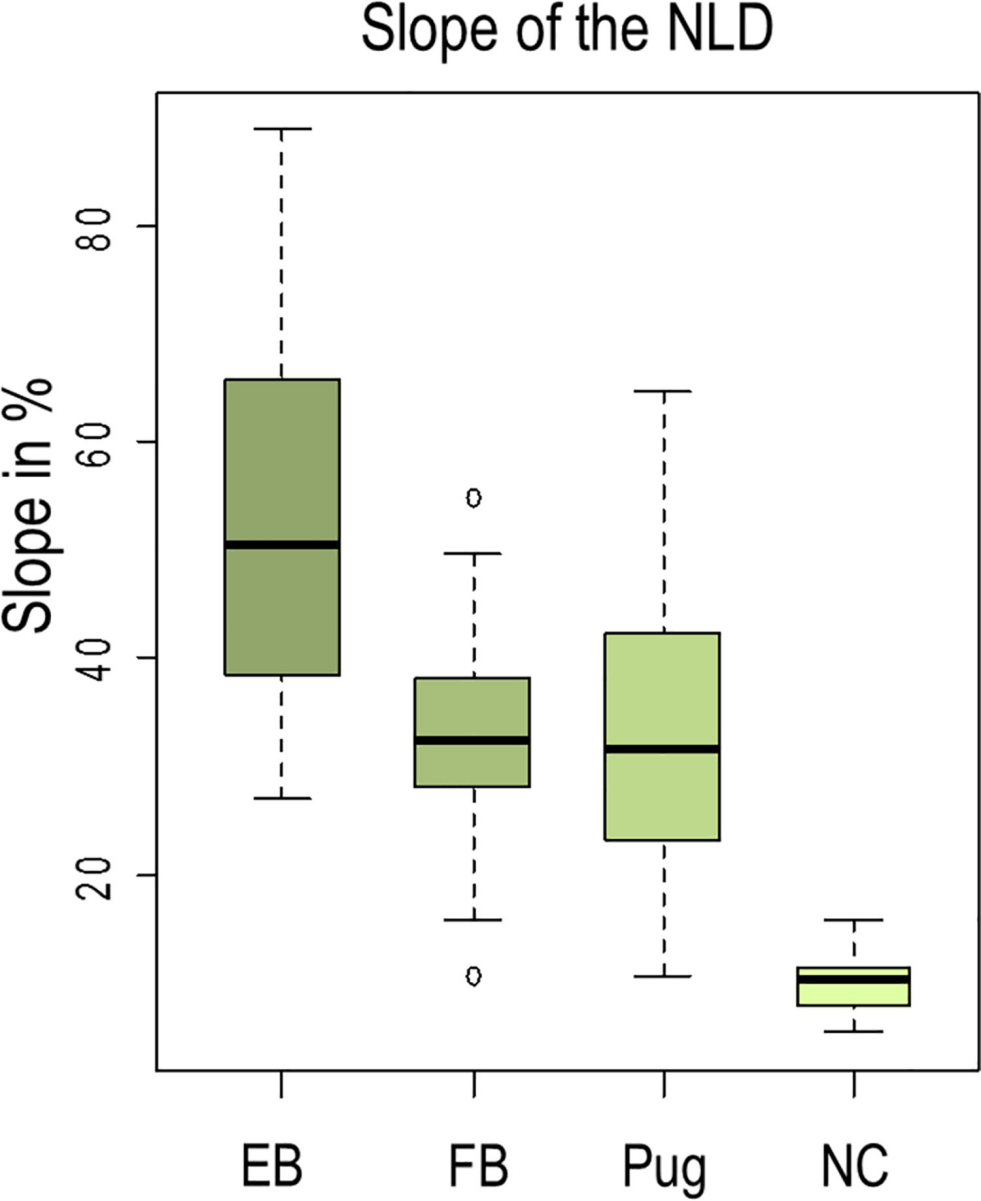
Gradient of the nasolacrimal duct (NLD). The slope in percent of the NLD in 10 English Bulldogs (EB), 18 French Bulldogs (FB), 20 Pugs, and six normocephalic dogs (NC) presented in a box-and-whisker diagram.

The NLD ascends in normocephalic dogs with a mean value of 10%, in Pugs with 32.5%, in French Bulldogs with 32.6%, and in English Bulldogs with 52.0%. Gradient determination requires particularly high image quality. Hence, we could not measure the ascent in all dogs. Detailed results of the slope measurement are listed in Table 1 and Fig 5.

**Table 1:** NLD gradient percentage in 10 English Bulldogs (bilaterally in all cases), 18 French Bulldogs (17 bilaterally, one unilaterally), 20 Pugs (18 bilaterally, two unilaterally), and six normocephalic dogs (bilaterally in all cases). The NLD of normocephalic dogs ascends slightly in comparison to the ducts of English Bulldogs, French Bulldogs and Pugs, which exhibit a steep slope.

	EB^a^ (n = 20)	FB^b^ (n = 35)	Pug (n = 38)	NC^c^ (n = 12)
Mean ascent % (range)	52.0 (27.0–88.8)	32.6 (10.7–54.8)	32.5 (10.7–64.6)	10.0(5.4–15.8)

^a^EB, English Bulldog

^b^FB, French Bulldog

^c^NC, normocephalic dogs.

### Angulation of the NDS

**Fig 6:**
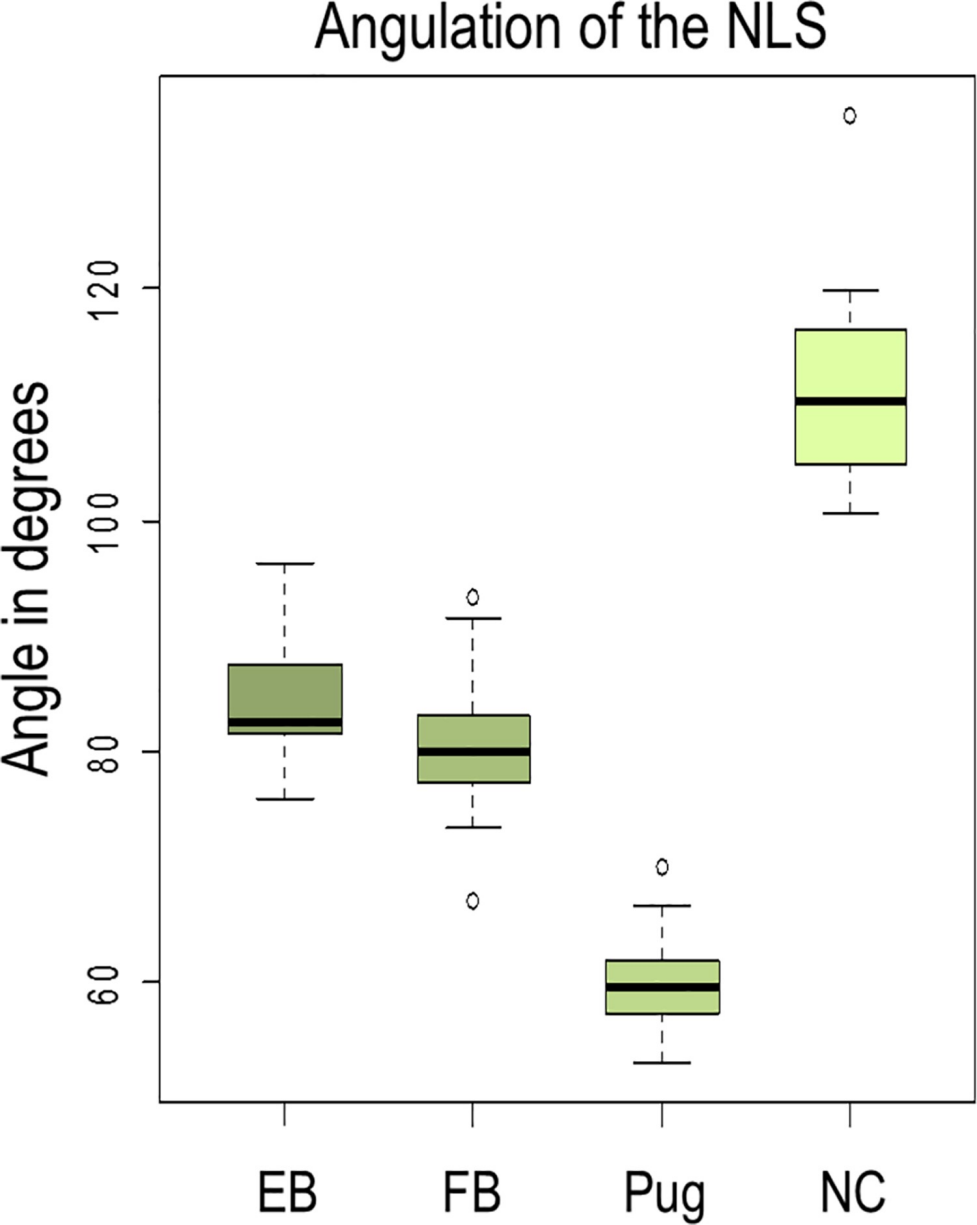
Angulation of the nasolacrimal system (NLS). The angle in degrees in 10 English Bulldogs (EB), 17 French Bulldogs (FB), 22 Pugs, and six normocephalic dogs (NC) presented in a box-and-whisker diagram.

**Fig 7:**
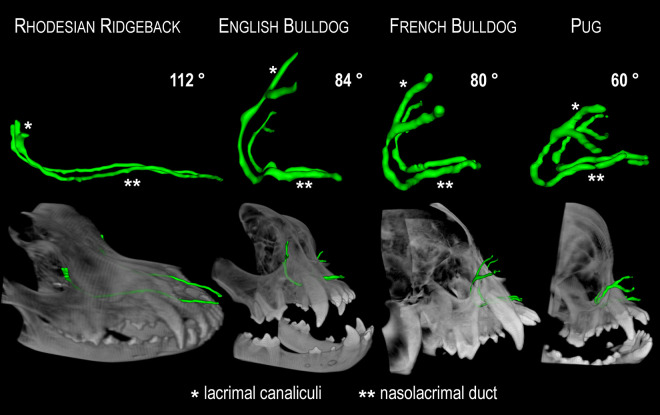
The different shapes of the tear drainage system (NDS) in normocephalic and brachycephalic dog breeds. The contrast-filled bilateral NDS, viewed from the side, is shown in green. At the top of the picture, it is shown outside its bony environment, while below it appears inside the skull. The given degrees reflect the angulation of the NDS. The NDS in normocephalic breeds has an obtuse-angled character, while brachycephalic dogs exhibit an acute-angled NDS, with the Pug showing the smallest values. These values support the depicted inversely directed course of the initial parts of the NDS in brachycephalic dog breeds. The very small angle in Pugs shows that the NDS is severely compressed in the vertical plane, with the lacrimal canaliculi and end portion of the nasolacrimal duct moving towards each other. In the lateral view, the characteristic shape of the NDS resembles the letter L in normocephalic and a U in brachycephalic dogs. In the case of a Pug, the compressed, clearly acute-angled appearance is more like a V than a U. Also noteworthy are the very long lacrimal canaliculi in brachycephalic dogs compared to the Rhodesian Ridgeback.

Measurements of NDS angulation revealed mean degrees of 112.0 in normocephalic dogs, 59.7 in Pugs, 80.4 in French Bulldogs, and 84.3 in English Bulldogs (Figs 6 and 7). The NDS in normocephalic breeds has an obtuse-angled character, while short-headed dogs exhibit an acute-angled tear drainage system, with the Pug showing the smallest values. The difference between the normo- and brachycephalic group is highly significant (p < 0.001). In the case of a Pug and a French Bulldog, the measuring points could not be determined with sufficient accuracy. The measured values are listed in Table 2.

**Table 2:** Angulation of the nasolacrimal drainage system in 10 English Bulldogs bilaterally, 17 French Bulldogs (15 bilaterally, two unilaterally), 22 Pugs (19 bilaterally, three unilaterally) and six normocephalic dogs (bilaterally in all cases). Measurements of NDS angulation revealed mean degrees of 84.3 in English Bulldogs, 80.4 in French Bulldogs, 59.7 in Pugs, and 112.0 in normocephalic dogs.

	EB^a^ (n = 20)	FB^b^ (n = 32)	Pug (n = 41)	NC^c^ (n = 12)
mean angulation in degrees (range)	84.3 (75.8–96.4)	80.4 (67.1–93.3)	59.7 (53.0–70.0)	112.0 (100.6–135.1)

^a^EB, English Bulldog

^b^FB, French Bulldog

^c^NC, normocephalic dogs.

### Length of NLD and lacrimal canaliculi

Nine of 18 French Bulldogs and nine of 23 Pugs fit the criteria for length measurements (good contrast across the entire NDS). The CT data of the English Bulldogs and normocephalic breeds could be completely included. The mean length of the NLD including the lacrimal sac is 94.0 mm in the normocephalic control group, in Pugs 40.1 mm, in French Bulldogs 43.7 mm, and in English Bulldogs 55.2 mm. The length of the NLD in brachycephalic dogs is substantially reduced compared with the normocephalic group. Nevertheless, their lacrimal canaliculi have much larger dimensions. Within the brachycephalic group, English Bulldogs have the shortest canaliculi in proportion to the entire length of the tear drainage system. Detailed values are summarized in Table 3.

**Table 3:** Lengths of the NLD and the inferior and superior lacrimal canaliculi (median values). The mean length of the NLD including the lacrimal sac is 55.2 mm in English Bulldogs, 43.7 mm in French Bulldogs, 40.1 mm in Pugs, and 94.0 mm in the normocephalic control group.

Length in mm	EB^a^	FB^b^	Pug	NC^c^
IC^d^ (range)	15.8 (11.8–22.7)	16.6 (14.2–20.2)	15.3 (11.9–19.0)	5.6 (2.9–7.7)
SC^e^ (range)	18.0 (14.3–2.2)	17.2 (12.5–20.4)	14.2 (11.0–17.4)	7.3 (5.3–12.3)
NLD^f^ incl. LS^g^ (range)	55.2 (48.8–69.9)	43.7 (39.0–49.0)	40.1 (31.5–44.5)	94 (76.4–116.7)

^a^EB, English Bulldog

^b^FB, French Bulldog

^c^NC, normocephalic dogs

^d^IC, inferior canaliculi

^e^SC, superior canaliculi

^f^NLD, nasolacrimal duct

^g^LS, lacrimal sac.

### Positional relationship of NLD and maxillary canine root

The present study showed that the NLD has to consistently cross beneath the misaligned maxillary canine root to reach the nasal cavity in Pugs (7/22; 31.8%) and French Bulldogs (11/18; 61.1%; Fig 3). One Pug could not to be evaluated because of insufficient image quality. In contrast, of the English Bulldogs, only one in ten (10%) exhibited this peculiar condition. However, even in Pugs and French Bulldogs whose NLD did not cross below the maxillary canine root, there was little space between the maxillary canines and the NLD. Specifically, in brachycephalic dogs, the initial part of the NLD inside the bony canal is in close contact with the maxillary canine roots and may even cross beneath them. In contrast, the NLD of normocephalic dogs reaches the nasal cavity long before its membranous middle portion passes by the maxillary canines. Because the NLD must cross beneath the canine root, it develops a steeper course. In the French Bulldog, the steepness of the NLD increased by 24,7% (3.8°) on the right side (p = 0.07), while it increased even more in the Pug: by 65.8% (9.2°) on the left (p = 0.012) and by 36.8% (5.7°) on the right side (p = 0.14). A statistically significant association could only be proven for the left side of the pug. It should be noted that non-parametric tests and medians (not mean values) were used for the calculation. It could not be proven that the crossing under the maxillary canine root directly impedes the drainage of fluid into the nasal vestibule (Chi-square test: right side p = 0.936, left side p = 0.501).

### Accessory opening of NLD

**Fig 8:**
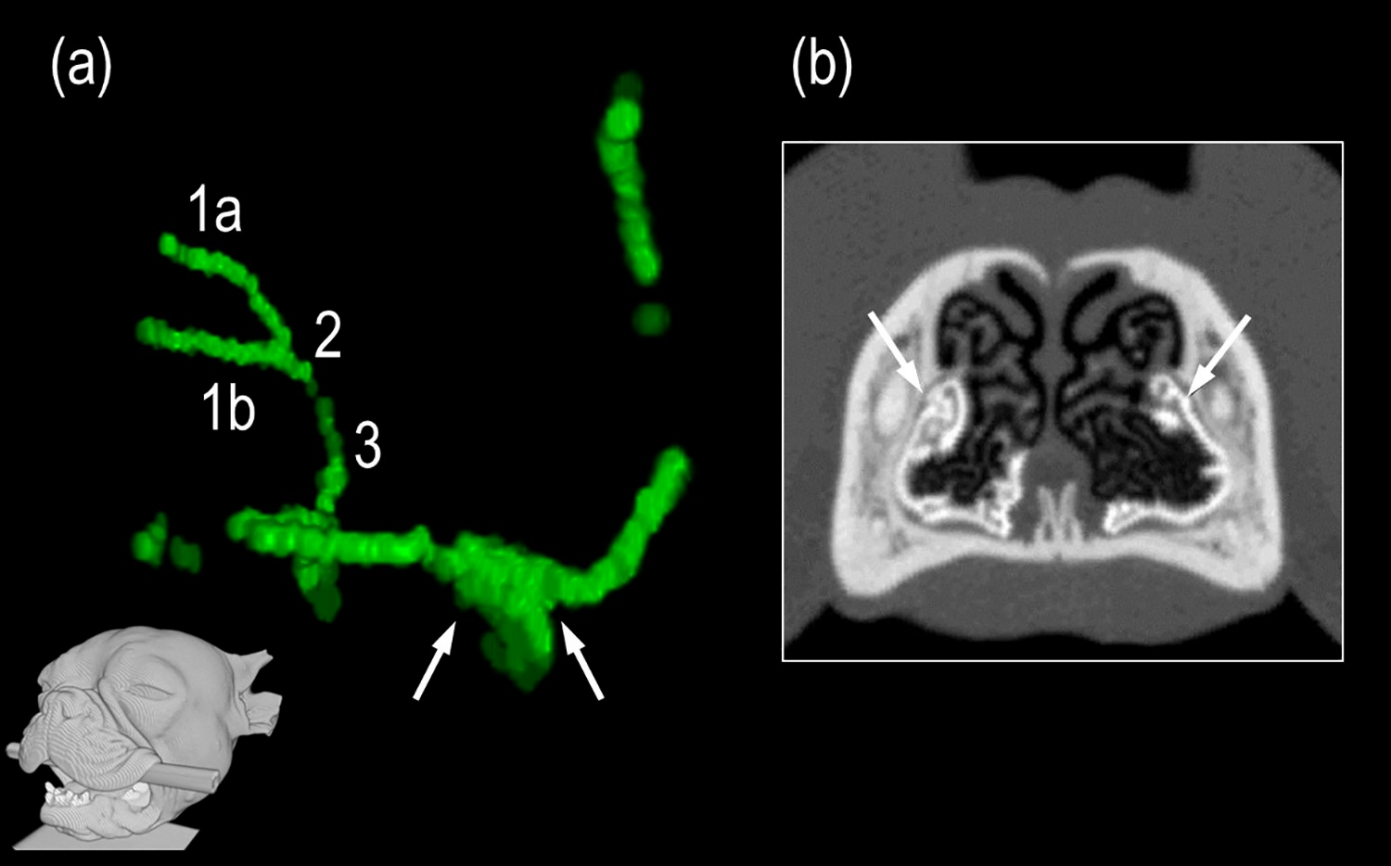
Accessory opening of the nasolacrimal system (NLS). (a) Three-dimensional illustration of the entire, contrast agent-filled bilateral NLS. The viewing perspective is represented by the head at the bottom of the picture. The white arrows point to the accessory opening of the left nasolacrimal duct. The contrast agent (shown in green) emerges from the opening. (1a) right upper lacrimal canaliculi, (1b) right lower lacrimal canaliculi, (2) right lacrimal sac, (3) right nasolacrimal duct. (b) Transverse CT-image of the nose. The white arrows point to the accessory opening, from which contrast agent emerges into the nasal cavity. From there, the contrast medium flows along the lateral nasal mucosa and collects on the floor of the nasal cavity.

An interruption of the NLD, the so-called accessory opening, was identified in all brachycephalic dogs except for one French Bulldog (98% of cases). It was typically located ventromedial to the maxillary canine root, shortly after the NLD exited its bony canal and merged into its membranous middle portion (Fig 8). Remarkably, in more than half of the dogs (54%), this additional orifice was the only detectable drainage route for the contrast agent into the nasal cavity. In those animals, no contrast agent left the NLD via the nasolacrimal ostium. In just seven of 50 cases (14%) was there an additional outflow via the ostium nasolacrimal. All six of the normocephalic dogs examined in this study showed an accessory opening similarly located level with the roots of the maxillary canines. However, in all these dogs, unlike the brachycephalic group, considerable quantities of contrast agent also escaped through the nasolacrimal ostia.

## Discussion

Brachycephalic dogs are affected by many breed-related disorders, some of them life-threatening. Selecting for questionable breed standards, such as an extremely short nose and flat face, are regularly associated with high-grade upper respiratory tract malformations [1, 16, 18–20]. Pronounced brachycephaly is also associated with ocular disorders. Exaggerated breeding for large eyes is accompanied by various anatomical deviations, such as an overly flat orbit, macroblepharon, lagophthalmos, entropion, and trichiasis [17, 21, 22]. Tear film deficiencies are also a common and serious problem [23, 24]. Even corneal sensitivity and nerve fiber density are disturbed [25, 26]. These malformations can lead to chronic painful conditions.

The present study showed that incorrect breeding and exaggerated emphasis on several external features leads to serious malformations in yet another organ system. Our results have demonstrated the extent of pathological changes in the tear drainage system in brachycephalic dogs. To our knowledge, the present study was the first to report these malformations in such detail. In addition, we showed that these changes are variable and breed-specific. The largest deviation from the normal physiological course occurred in the Pug.

To date, the literature has shown that the course of the NDS differs considerably between normo- and brachycephalic animals [3, 14, 15]. For example, in brachycephalic cats, the steep course of the NLD is thought to impede proper tear drainage, causing watering eyes, which are an almost universal finding in these cats [3, 13]. Several anatomical peculiarities result in an acute-angled NLD alignment in these cats, namely severe shortening of the facial skull, most notably the nasal bones, accompanied by an aberrant orientation of jaw, palate, and nose, which are all dorsally displaced [13].

According to the present study, the NDS of brachycephalic dogs exhibits a distinctive course that varies substantially from that of normocephalic dog breeds and shows several similarities to that of brachycephalic cats [3]. In contrast to normocephalic dogs, the NDS of short-headed dogs, starting at the lacrimal puncta, initially follows an inverse direction, before turning rostrally. This peculiar course results from the rostrally displaced lacrimal canaliculi, caused by the forward position of the globes in brachycephalic dogs [17]. Furthermore, the first part of the NLD is translocated posteriorly due to severe midface shortening. The difference in NDS angles between the normo- and brachycephalic groups corroborate these findings. The acute-angled shape in brachycephalic dogs is inevitably associated with rostrally displaced lacrimal canaliculi and/or parts of the NLD, unlike the obtuse-angled NDS in normocephalic dogs. In particular, the extremely small angle in Pugs results in severe NDS compression in the vertical plane, so that the lacrimal canaliculi and end portion of the NLD move closer together. Again, this mirrors the very small distance between the eyes and nose in the Pug [27].

Furthermore, the extreme reduction of nasal structures in brachycephalic dogs is accompanied by a shortened NLD, while their lacrimal canaliculi are greatly elongated (Figs 4 and 7). In normocephalic breeds, the short canaliculi account for as few as 5%-8% of the entire length of the lacrimal system, while they comprise 17%-35% of the length in brachycephalic dogs. Specifically, a 1972 study [10] revealed 4–5 mm long canaliculi and a 25–30 mm long NLD in brachycephalic dogs. In the present study, the lengths of the canaliculi exceed these values by two- to threefold, with the English Bulldog exhibiting canaliculi 12–23 mm long, the French Bulldog showing canaliculi 13–20 mm, and the Pug displaying 11–19 mm-long canaliculi. Considering the protruding ocular globes in modern brachycephalic dog breeds, which have to be enclosed by their eyelids, the values are not likely to be much lower. Unfortunately, no detailed information is available about the short-headed breeds examined in the 1972 study. The lengths of the lacrimal canaliculi of normocephalic dogs in the present study are similar to those found in previous studies; that is, between 4 and 7 mm [10, 11]. The length values of the NLD in the brachycephalic groups of the present study (25–30 mm) also exceeded those of the 1972 study. These rather long canaliculi contribute greatly to forming the peculiar U- or V-shaped NDS that occurs in brachycephalic dogs. The lengths of the NLD in the normocephalic dog breeds examined in our study were overall slightly above the previously reported values of between 35 and 100 mm [10]. We measured a length of 76 to 117 mm. The included length of the lacrimal sac in our study was of nearly no consequence, as it only measured between 2 and 5 mm long [11]. Importantly, the NLD in brachycephalic dog breeds was shortened by half compared with normocephalic dogs of similar size, and their lacrimal canaliculi lengthen by two to three times. Of particular interest is the steeper NLD course in the short-headed dogs examined in the present study, as has already been described in the brachycephalic cat [3, 15]. The measured gradient values of brachycephalic dogs averaged between 32.5% and 52.0%, exceeding those of the normocephalic group by far (10.0% on average). As in brachycephalic cats [3, 13], the NLD consistently had to cross beneath the maxillary canine root to reach the nasal cavity in Pugs and French Bulldogs. This led to a steeper NLD course by 25%-66%. In the cat, this “uphill” course distinctly interferes with tear drainage and results in chronic epiphora [3, 13, 14]. However, in the brachycephalic dog, the malformation did not interfere with complete removal of tears from the ocular surface. The key difference was an abnormal drainage mode. In short-headed dogs, there was consistently an accessory opening that facilitated complete tear outflow into the nasal cavity, regardless of NLD steepness. The steep alignment influences flow dynamics in the NLD, mainly behind the accessory opening–i.e., in the middle and end portions of the NLD. Such an additional opening was seen in all brachycephalic dogs examined, except for one French Bulldog (98% of cases). In all brachycephalic dogs, this orifice comprised the main outflow pathway for the contrast medium into the nasal cavity. In fact, it was the only detectable pathway in more than half of the animals (54%). This seems to be the path of least resistance to tears, because the opening occurs at the beginning of the slope. Even if tear fluid continues to flow forward, it does so in very small quantities. This implies that the ostium nasolacrimale and thus, also the middle and end portion of the NLD play only a subordinate role in tear drainage processes in brachycephalic dogs. Contrary to this, all the normocephalic dogs showed drainage of contrast agent via both the accessory opening and the nasolacrimal ostium. The reported prevalence of an accessory opening in dogs has varied between 40% [11] and 90% [12] in previous literature. In one previous investigation, all but one of seven brachycephalic dogs had such an additional opening [12]. No accessory opening has been found in cats [14, 15]. In conclusion, although there were several parallels, the situation differs significantly between brachycephalic dogs and cats. The steep alignment of the NLD in cats is associated with severe impairment of tear drainage. In contrast, the NLD in brachycephalic dogs remains functional, despite having a similar steepness, because it mainly drains into the posterior nasal cavity just before the steep ascent begins (Fig 8). If no such accessory opening occurred, it is likely that brachycephalic dogs would experience the same tear drainage problems as brachycephalic cats.

These aberrant conditions in brachycephalic dogs are of additional significance with regards to testing the patency of the NDS in daily practice. Brachycephalic animals are often presented to veterinarians due to epiphora. Usually, the clinicians suspect that the animals have a problem with their NDS. Commonly used tests, such as the primary Jones test and flushing of the NDS with saline or diluted povidone iodine, are considered positive when the applied agent exits the nostrils [28, 29]. In brachycephalic dogs, the NDS leaks into the posterior nasal cavity instead of the nasal atrium, so the primary Jones test has no or little diagnostic value in such breeds. In fact, it can lead to misinterpretations and inadequate treatment. To return to epiphora, a wide range of factors cause watering eyes in short-headed dog breeds. Unlike in brachycephalic cats, epiphora is not caused by a steep NLD. It is known from the literature, that a complex of breed-linked anatomical and functional ocular disorders play an important role, many of which are treatable or improvable, especially macroblepharon, tear film abnormalities, or trichiasis [28, 29]. The situation ultimately differs from that in brachycephalic cats. Therefore, epiphora should not be dismissed from the outset as an anatomical problem of the NDS in brachycephalic dogs. The extent to which malformations of other areas of the NDS impair its function and thus promote epiphora is not conclusively clarified in this study.

The problems discussed above raise the question of whether lacrimal drainage into the posterior nasal cavity is associated with any adverse effects. Short-headed dogs often have a remarkably dry and hyperkeratotic nasal planum, and no previous studies have addressed the possible connection between the lack of tear fluid in the nasal vestibule and hyperkeratosis in brachycephalic dogs, to the knowledge of the authors. According to Williams (2008), the nasal glands are mainly responsible for humidifying the nasal entrance, while tear fluid has subordinate importance. However, recent studies have shown that the fluid of the lateral nasal gland does not reach the vestibule but flows caudally into the nasal cavity [6]. Perhaps the "loss" of tear fluid via the accessory opening is causally involved in hyperkeratosis of the nasal planum in brachycephalic dogs.

It is also unclear whether the underdeveloped lacrimal sac in brachycephalic dogs, together with the altered canaliculi, disturbs the lacrimal pump mechanism [30–33] and thus represents a permanent drainage obstruction.

The main limitation of the present study was that the behavior of contrast agent within the NDS of anesthetized dogs may not reflect the real-world flow dynamics of tear fluid. On the one hand, the contrast agent was applied using a catheter, while on the other, it is much more viscous than tear fluid. However, in the authors’ experience, fluid consistently escapes via the accessory opening in the primary Jones test, and through flushing of the NLD in conscious brachycephalic dogs. Consequently, it can be presumed that there are only minor differences between the conditions shown in our study and real flow dynamics.

## Conclusion

This study shows a further consequence of exaggerated breeding for a short head conformation. The NDS is grossly malformed in all three brachycephalic breeds. The length of the NLD is substantially reduced and it exhibits a steep course, the angulation is severely compressed. The lacrimal canaliculi have much larger dimensions, follow an inverse direction and are displaced rostrally.

Obviously, clinical symptoms remain absent only because all animals examined had an aberrant outflow pathway through the accessory opening. Whether this abnormal drainage and thus an absence of this fluid in the nasal vestibule is related to the often remarkably dry and hyperkeratotic nasal planum of short-headed dogs can be speculated and may be answered in further studies.
